# Supplementary material for: Rapid turnover of life-cycle-related genes in the brown algae
Source: Genome Biol. 2019 Feb 14;20:35. doi: 10.1186/s13059-019-1630-6 (PMC6374913; doi:10.1186/s13059-019-1630-6)
Supplement: Supplementary file 3 — Protein alignments, corresponding Gblocks html file and CDS sequences for the lineage conserved gametophyte-biased genes. (ZIP 2109 kb) [file 13059_2019_1630_MOESM3_ESM.zip › Con_GA_alignments/Ec-14_004890.aa_ali.fasta-gb.htm]

Ec-14\_004890.aa.fasta.trans.aa\_ali.fasta


## Gblocks 0.91b Results

Processed file: **Ec-14\_004890.aa.fasta.trans.aa\_ali.fasta**  
Number of sequences: **2**  
Alignment assumed to be: **Protein**  
New number of positions: **669** (selected positions are underlined in blue)

```
                         10        20        30        40        50        60
                 =========+=========+=========+=========+=========+=========+
Ec-14_004890     MSSRCGQPVVSSSSQRGCLRLEASAAPQQQRRPKPRKIPCPNRPSLSRKATTTTSTAGSA
MSTRG.50872.1    ------MVVVASSSRHNGHRPPSRPRKGLRSSSDTAGSIADTTAATAGVAPSSSSSKSNS
                         ############                                        


                         70        80        90       100       110       120
                 =========+=========+=========+=========+=========+=========+
Ec-14_004890     ETLNRRRTKS---DN-ASKLQELKLAFGLLSKEIQGLDGEEGDQCLPCPPATESSAAVTK
MSTRG.50872.1    IIIDNHNNDHNNNDNRNPKLQELQVAFCLLSKQIQGLDGE-GNHVLTCPNPPVPAAQRTD
                                   ###################### ################## 


                        130       140       150       160       170       180
                 =========+=========+=========+=========+=========+=========+
Ec-14_004890     GEMRRNEASKPLTPAVKNTGIPPSAVATEEAPQQSHGGLAAAANLPAKKAATSHPGVQQR
MSTRG.50872.1    ----PLESPALITPPTEATGSKPQRLLDSRL-------TVSSAVVPGEEAGTSRARVRER
                       #################                   ##################


                        190       200       210       220       230       240
                 =========+=========+=========+=========+=========+=========+
Ec-14_004890     SNEAADDLNRMGADQRNKTAADGLDRVRASSHAEGRKAAEAA-AKQAKQSLKQLDLELDK
MSTRG.50872.1    VEA----------------ARDSVRKSKPISHVEGRNAVEPIVPERGQQDLRLLRRELEA
                                    #####################        ############


                        250       260       270       280       290       300
                 =========+=========+=========+=========+=========+=========+
Ec-14_004890     ERRARTAAESTLHQLQLRATESDLEREKLERVLASFGGRSLQEITTASQLLERRLKEAET
MSTRG.50872.1    EKRSRIAAESALQQLQRRAAESALERERLERVVNSFGGRSLEELSTRLQATERRMREAEA
                 ############################################################


                        310       320       330       340       350       360
                 =========+=========+=========+=========+=========+=========+
Ec-14_004890     ALVNSQQLTAAKAAEGLEALEQAASLAEQLETERVARAAAEEARRSVTQELRCCLRGRAN
MSTRG.50872.1    ALAPSQHLAAEKAAESLEALEQAASLAESLETERTARIAAENEKRTVTQELRCCLRGRAK
                 ############################################################


                        370       380       390       400       410       420
                 =========+=========+=========+=========+=========+=========+
Ec-14_004890     AEQQARAATAALKKSEAQRESLAQRLEVLRTANKRLDKRVAAVALQSSVGAR------AR
MSTRG.50872.1    AEQEAKAARGATKKSEAQRDSLTQRLEILRTANRRLEKRAAAVALPASFAAGTGIDIVGS
                 ###################################################         


                        430       440       450       460       470       480
                 =========+=========+=========+=========+=========+=========+
Ec-14_004890     SNAGGGGGATDDSVARLAAARARLKVLSTSGAAVSLNNNTGKG------RGQSHAGEERA
MSTRG.50872.1    GNGGCDGDSAGNTAARLLTARARLKVLSASGRSSVMGSNAGVWGHPREHDGEIEACKEKA
                  ########################################         ##########


                        490       500       510       520       530       540
                 =========+=========+=========+=========+=========+=========+
Ec-14_004890     AVERAVGTVAAEKQWRDKLRNAEMQGEVFQSAAKASATGMKIALKQKAELEEMNRVLVKQ
MSTRG.50872.1    AIERAISNAVGEKRWRDKLRRAEMQGEAFQSAAIAAETGLKIAVRQSAELEEVTRILAKK
                 ############################################################


                        550       560       570       580       590       600
                 =========+=========+=========+=========+=========+=========+
Ec-14_004890     LAGDMPAEERELIGRMADDEDVIR--SPAAPLVVGTD---GESTYRDLGKDETTATLVPR
MSTRG.50872.1    LAHDKYDTKRGSIVQIGTTSNAAGGKRPVSLLAAGDDDVENTTDTPEGGGAERARTCSTP
                 #############              ##########                       


                        610       620       630       640       650       660
                 =========+=========+=========+=========+=========+=========+
Ec-14_004890     VLGSRIESERQHHASATQCSSAVSYP----------AVDRSSGLPAGDG---ATGETETA
MSTRG.50872.1    GIPAPVAEREGQPSVPEQCCRTASFPPPPILLPDTEQVTDATGKAIASGGKPACSTGEVC
                                                      ############   ########


                        670       680       690       700       710       720
                 =========+=========+=========+=========+=========+=========+
Ec-14_004890     ATPTPLRPPDNNPEMERNHNQQPTEPCNRSRQEGGK--ETTMPPSPPCVIPQKLRPAAVR
MSTRG.50872.1    GPALPEKETDRDALTGNDPATQTSQHLERGCALGEVERRRQMPPTPHGSGCQAS-VSSTT
                 ##########           #############       ###########        


                        730       740       750       760       770       780
                 =========+=========+=========+=========+=========+=========+
Ec-14_004890     SYSVDACRSGATRTTSPWRHDGCPSRPRSASASANTSPSPCTTTPTKVRCEPQQQRHHHQ
MSTRG.50872.1    IESVSECKS-GTRTTPPSPVEKSCLGHQSRSRSASVGEVPTLSTRRCGIMAPQAQLQHHE
                                             ############################### 


                        790       800       810       820       830       840
                 =========+=========+=========+=========+=========+=========+
Ec-14_004890     EGDGMVVARNNDACCCSSSAAPFNQLFFESKKLRDEVLSAVRNGPLCWEETRCKACSGGN
MSTRG.50872.1    QEQHQRHTHRHHRQQHPQQKSPR-------QQLHEEAVSLMGGTNCCSPRMTAVPSSTPL
                                                 ###############             


                        850       860       870       880       890       900
                 =========+=========+=========+=========+=========+=========+
Ec-14_004890     GSGNGSGDVGVGVGVGVGGAGGGDAGGGSGGRG-GGSDGDGGYSGSQLEKVGGSTAVTSV
MSTRG.50872.1    SESRRMREEVLSTERNCAYGSNLQASSGEDDFGSGGGCGREGWRKLRQENAQHQSTVASV
                                                   ##########################


                        910       920       930       940       950       960
                 =========+=========+=========+=========+=========+=========+
Ec-14_004890     PIEVLLRLCDRGARKAWRKREGGVADCAVG--GEKPIAGGVAANQDLDREGMADERSDRQ
MSTRG.50872.1    PIEVLLRLCDRSVRGTRCEKEAAYSVCSGDRTGCSDPGKDPTANQSADEVGGKEDTKSRD
                 ###########################               ##################


                        970       980       990      1000      1010      1020
                 =========+=========+=========+=========+=========+=========+
Ec-14_004890     NLTESPAEGSKITLDDDATHLTSPLPPGITRALAQQVQGAVARGDERAFLNIVGRCGSTA
MSTRG.50872.1    HGVEQPAKQVAFPATDNAARPAVPLPPGISPTLAQQVQGAVSRGDERGFLEIIERCGSTE
                 ############################################################


                       1030
                 =========+====
Ec-14_004890     REGIAKLLAINFPE
MSTRG.50872.1    KGGIARLLSIGAPE
                 ##############
```

```
Parameters used
Minimum Number Of Sequences For A Conserved Position: 2
Minimum Number Of Sequences For A Flanking Position: 2
Maximum Number Of Contiguous Nonconserved Positions: 8
Minimum Length Of A Block: 10
Allowed Gap Positions: None
Use Similarity Matrices: Yes
```

```
Flank positions of the 18 selected block(s)
Flanks: [9  20]  [79  100]  [102  119]  [127  143]  [163  180]  [200  220]  [229  411]  [422  461]  [471  553]  [568  577]  [638  649]  [653  670]  [682  694]  [702  712]  [749  779]  [813  827]  [875  927]  [943  1034]  

New number of positions in Ec-14_004890.aa.fasta.trans.aa_ali.fasta-gb:  669  (64% of the original 1034 positions)
```
